# Species-specific ecological niche modelling predicts different range contractions for *Lutzomyia intermedia* and a related vector of *Leishmania braziliensis* following climate change in South America

**DOI:** 10.1186/s13071-017-2093-9

**Published:** 2017-03-24

**Authors:** Shannon McIntyre, Elizabeth F. Rangel, Paul D. Ready, Bruno M. Carvalho

**Affiliations:** 1 Laboratório Interdisciplinar de Vigilância Entomológica em Diptera e Hemiptera, Instituto Oswaldo Cruz, Fundação Oswaldo Cruz, Rio de Janeiro, Brazil; 2 Department of Disease Control, Faculty of Infectious Tropical Diseases, London School of Hygiene and Tropical Medicine, Keppel Street, London, WC1E 7HT UK

**Keywords:** *Lutzomyia intermedia*, *Lutzomyia neivai*, Ecological niche modelling, Range changes, Climate change, Cutaneous leishmaniasis, South America

## Abstract

**Background:**

Before 1996 the phlebotomine sand fly *Lutzomyia neivai* was usually treated as a synonym of the morphologically similar *Lutzomyia intermedia*, which has long been considered a vector of *Leishmania braziliens*is, the causative agent of much cutaneous leishmaniasis in South America. This report investigates the likely range changes of both sand fly species in response to a stabilisation climate change scenario (RCP4.5) and a high greenhouse gas emissions one (RCP8.5).

**Methods:**

Ecological niche modelling was used to identify areas of South America with climates currently suitable for each species, and then the future distributions of these climates were predicted based on climate change scenarios. Compared with the previous ecological niche model of *L. intermedia *(*sensu lato*) produced using the GARP algorithm in 2003, the current investigation modelled the two species separately, making use of verified presence records and additional records after 2001. Also, the new ensemble approach employed ecological niche modelling algorithms (including Maximum Entropy, Random Forests and Support Vector Machines) that have been widely adopted since 2003 and perform better than GARP, as well as using a more recent climate change model (HadGEM2) considered to have better performance at higher resolution than the earlier one (HadCM2).

**Results:**

*Lutzomyia intermedia* was shown to be the more tropical of the two species, with its climatic niche defined by higher annual mean temperatures and lower temperature seasonality, in contrast to the more subtropical *L. neivai*. These different latitudinal ranges explain the two species' predicted responses to climate change by 2050, with *L. intermedia* mostly contracting its range (except perhaps in northeast Brazil) and *L. neivai* mostly shifting its range southwards in Brazil and Argentina. This contradicts the findings of the 2003 report, which predicted more range expansion. The different findings can be explained by the improved data sets and modelling methods.

**Conclusions:**

Our findings indicate that climate change will not always lead to range expansion of disease vectors such as sand flies. Ecological niche models should be species specific, carefully selected and combined in an ensemble approach.

**Electronic supplementary material:**

The online version of this article (doi:10.1186/s13071-017-2093-9) contains supplementary material, which is available to authorized users.

## Background

The Intergovernmental Panel on Climate Change (IPCC) estimates that annual mean surface temperature has risen throughout the South American continent since 1901, and will continue to do so over the coming century [1]. These changes are anticipated to alter the distribution and risk of contracting vector-borne diseases, due to the impact of bioclimatic conditions on the development, behaviour and lifespan of many insects [2]. Climatic conditions are cited as amongst the most important factors influencing the density and the number of annual generations of the sand fly species (Diptera, Phlebotominae) transmitting *Leishmania* species (Kinetoplastida, Trypanosomatidae) that cause human leishmaniasis [3, 4]. The present report uses ecological niche modelling [5] to define the current distributions of two leishmaniasis vectors in South America, *Lutzomyia* (*Nyssomyia*) *intermedia* (Lutz & Neiva, 1912) and the closely-related *Lutzomyia* (*Nyssomyia*) *neivai* (Pinto, 1926), and to predict their geographical ranges in 2050 under two climate change scenarios, Representative Concentration Pathway (RCP) 4.5 and RCP 8.5, both based on the HadGEM2-ES climate model [1].

Before 1996, *L. neivai* was usually treated as a junior synonym of the morphologically similar *L. intermedia* [6], which has long been considered an important vector of *Leishmania braziliens*is, the causative agent of much cutaneous leishmaniasis (CL) in South America [7, 8]. Both sand fly species are now incriminated vectors of *L. braziliensis* [4, 9] in different regions, such as *L. intermedia* in south-east Brazil [10] and *L. neivai* in south Brazil [11] and Argentina [12]. Nevertheless, many earlier records do not permit the differentiation between the two species and previous authors have not recognised them as separate species. In those cases, we refer here to *L. intermedia *(*sensu lato*). The females of *L. intermedia* and *L. neivai* are opportunistic blood feeders, feeding on domestic animals, rodents and humans alike, and can be found in both forests and anthropic environments in Argentina, Bolivia, Brazil or Paraguay [13–15]. Distinguishing between *L. intermedia* and *L. neivai* is important because any differences in their habitat preferences, adaptations to deforestation and urbanisation, biting preferences and vectorial capacities could influence which areas are at risk of leishmaniasis transmission [13].

Ecological niche modelling has emerged in recent years as a key method for predicting the potential distribution of a species [5]. Ecological niche models have already been constructed for several sand fly species in parts of Latin America, with or without predictions based on climate-change scenarios [16–22]. Ecological niche modelling on a continental scale has only been reported for *L. intermedia *(*s.l*.) [16] because of the earlier paucity of verified presence records for *L. intermedia* and *L. neivai*. A published niche model of *L. neivai* is restricted to north-west Argentina because it is based on field-collected data [18]. However, datasets and methods are frequently being improved [19, 23–25], and the present report is the first to investigate how differences in the fundamental ecological niches of *L. intermedia* and *L. neivai* could affect their future distributions throughout South America.

## Methods

### Study area

All reviews from 1978–2007 record *L. intermedia* and *L. neivai* from just four countries, namely Argentina, Brazil, Bolivia and Paraguay [13, 26–28]. However, our study area included all countries in South America, to investigate the potential range of both species.

### Presence records for the two sand fly species from all countries

#### Sources

To compile records of the presence of *L. intermedia* and *L. neivai* in South America, the online databases PubMed, ISI, Scopus and SciElo were searched on 18^th^ July 2016 for relevant studies using the terms ‘*Psychodidae*’ and ‘*Lutzomyia*’. Recovered papers were scanned for mention of *L. intermedia* and *L. neivai* in the context of entomological surveys, and all records compiled in a Microsoft Excel database (see Additional file 1: Table S1). Additionally, the presence lists compiled by Martins et al. [26], Marcondes [29] and Andrade-Filho et al. [13] were consulted to ensure any other unique presence records were not missed. *Lutzomyia* (*Nyssomyia*) *intermedia*, *Lutzomyia* (*Nyssomyia*) *neivai*, *Lutzomyia intermedia*, *Lutzomyia neivai*, *Psychodopygus intermedius*, *Nyssomyia intermedia*, and *Nyssomyia neivai* were all considered valid species names.

Between February 2014 and July 2016, BMC also checked for additional, unpublished presence records of the two species by contacting Brazilian Health Department registers and performing physical searches of the entomological collections of the Brazilian institutes Centro de Pesquisas René Rachou (FIOCRUZ, Belo Horizonte, assisted by Dr J. D. Andrade-Filho), Instituto Butantan (IBUT, São Paulo, assisted by Dr R. Moraes), Instituto Evandro Chagas (IEC, Belém, assisted by Dr T.V. Dos Santos), Instituto Oswaldo Cruz (FIOCRUZ, Rio de Janeiro, assisted by Dr J. M. Costa), Instituto de Pesquisas da Amazônia (INPA, Manaus, assisted by Dr R. Freitas and Dr M. L. Oliveira), Universidade de São Paulo Faculdade de Saúde Pública (USP, São Paulo, assisted by Prof. E. Galati and Prof. M. A. Sallum), and Universidade de São Paulo Museu de Zoologia (data provided by Dr A. J. Andrade).

#### Inclusion-exclusion criteria

Presence records gathered from sources prior to 1996 were cross-referenced with the major reviews of Marcondes et al. [28] and Andrade-Filho et al. [13], to check for any inconsistencies in the identifications of *L. intermedia* and *L. neivai*, which were reclassified when necessary. All presence records were plotted on a map of South America using ArcGIS v. 10.0 [30] to identify potential outliers. If a record appeared to fall outside the distributions of *L. intermedia* and *L. neivai* described by the two major reviews [13, 28], the original paper was consulted to assess the accuracy of the database entry and the taxonomic expertise of the identifier. The authors were then contacted for verification, and expert opinion was sought from those listed in the previous section.

#### Data preparation for modelling

Presence records from secondary data tend to be spatially biased; therefore, the datasets were refined to reduce spatial autocorrelation. A spatial thinning process was applied with R package *spThin* [31], to randomly select a subset of records for which each neighbouring pair was at least 10 km apart. The remaining data was retained for independent validation of the models.

Pseudo-absences were sampled outside the environmental domain favourable for the species [32], with the latter estimated using the simple bioclimatic envelope model BIOCLIM [33]. The number of pseudo-absences was the same as the number of presence records for each species.

A dataset of presence records published before 2002 was created for comparison of our results with the previously published ecological niche model of *L. intermedia *(*s.l*.) [16].

### Climatic variables and climate change projections

#### Sources

Historical (1960–1990) climate data for South America was sourced from WorldClim, an online database of 19 climatic variables derived from monthly averages of temperature and precipitation [34]. Also sourced from WorldClim were climate projections for 2050 (average for 2041–2060) under different scenarios based on the different RCPs underlying the IPCC’s Fifth Assessment Report models. Each of the RCPs is based on potential increases in total radiative forcing (defined as the ‘cumulative measure of human emissions of [greenhouse gases] from all sources expressed in Watts per square meter’), simulated in integrated assessment models to 2100 [35]. RCP4.5 and RCP8.5 were chosen because they represent contrasting greenhouse gas emissions scenarios. RCP4.5 is a stabilisation scenario [36]. It assumes growth in the greenhouse gas emissions trajectory is limited through initiatives including carbon capture and storage, the development of low emissions energy technologies, and the introduction of global greenhouse gas emissions pricing. RCP8.5 corresponds to the highest greenhouse gas emissions scenario in the RCP collection. It is a ‘business as usual’ scenario, in which no climate-specific mitigation targets or policies are set, population growth is high, and only modest improvements in energy-use intensity and technology change are experienced [37]. It does assume a slight reduction in emissions intensity from the 2010 baseline after 2030.

Downscaled and calibrated projections of the HadGEM2-ES model were selected because they have demonstrated good predictive ability for climate in South America [38]. Two and a half minute spatial resolution (approximately 25 km^2^ per pixel) was chosen for all bioclimatic variables, which is an adequate resolution for ecological niche models based only on climate variables [39].

#### Selection of climatic variables

A subset of variables was selected, to reduce collinearity in the dataset of 19 climatic variables. A Pearson correlation matrix was applied to identify pairs or groups of highly-correlated variables (*r* > 0.6) and, with one exception, all removals were based on a selection criterion of ecological relevance to the vector. The final set of climate predictors used to run the ecological models consisted of annual mean temperature (BIO1), mean diurnal range of temperature (BIO2), temperature seasonality (BIO4), annual precipitation (BIO12), precipitation seasonality (BIO15) and precipitation of warmest quarter (BIO18) [34].

### Description of sand fly climatic niches

The values of the climatic variables for each presence record were extracted and compared statistically and by constructing scatter plots, using ArcGIS v. 10.0 [30] and R [40], to describe any differences in the niches of *L. intermedia* and *L. neivai.* Statistical significance was assessed by Wilcoxon rank sum tests in R.

### Ecological niche modelling

#### Model selection

There are numerous approaches to ecological niche modelling, with each algorithm producing a different predictive result and map. The choice of algorithms will depend in part on the availability of presence data alone, presence and background data (the environment across the entire study area), or both presence and absence data. However, there is no single approach that is consistently considered superior to all others, as discussed by Araújo et al. [41], Hijmans & Graham [42] and Beaumont et al. [43] in relation to predicting species distributions under climate change. Therefore, to overcome the limitations of each algorithm when used in isolation, five modelling algorithms were applied: BIOCLIM, Generalised Linear Models (GLM), Maximum Entropy (MaxEnt), Random Forests (RANFOR), and Support Vector Machines (SVM).

BIOCLIM is classified as a ‘profile’ modelling method, i.e. it only considers species presence data [5]. It works by computing the similarity of environmental variables at known locations of species occurrence to the value of those variables at locations where the species has not been observed, to identify potentially climatically suitable environments [5, 33, 42]. GLMs are a form of regression model. In this method, the dependent variable is transformed relative to its mean value, and the relationship between the transformed variable and a set of predictor variables assessed to forecast climatically suitable environments for a species [44]. Logistic regression was utilised for this study because it is the most popular form of GLM for ecological niche modelling and adequate for presence/absence data.

MaxEnt, RANFOR and SVM are all machine learning models that consider both presence and absence or presence and background data [5]. MaxEnt computes a probable distribution within the study area that satisfies constraints derived from the environmental conditions at current presence locations. It then selects an area that has maximum entropy within the specified distribution area [45]. RANFOR is a classification tree-based modelling method that works by dividing the data into homogeneous subgroups based on the value of predictor variables and describing each subset resulting from these splits according to their homogeneity in the response variable through a sum of squares [46]. SVM models estimate the current and future fundamental niche of a species by fitting a hyperplane to separate presence and absence data, and applying a linear analysis [46]. Among machine learning and other modelling algorithms, MaxEnt models have consistently performed well in comparative and validation studies when used to predict habitat changes due to climate change [5, 42].

The algorithm GARP [47] was used to model the ecological niche of *L. intermedia * (*s.l*.) to help compare our findings with those of Peterson & Shaw [16]. GARP is a genetic algorithm based on a series of classificatory rules that are developed according to relationships between predictive variables and species occurrences. The various rules 'evolve' in a process analogous to natural selection, and they are excluded or selected to maximise predictability [46].

#### Model settings and evaluation

Models were run in R package *dismo* [5] under default settings, except for GARP models that were run in OpenModeller (version 1.1.0), using its 'Best Subsets' implementation [48]. For every modelling algorithm, 10-fold cross-validation was applied, to use the whole set of presence/pseudo-absence records for both model training and testing. In each model run, 10% of records were randomly selected for model testing. 60 model runs were performed; 10 runs for each of the six algorithms.

The model outputs were mapped as continuous values per pixel representing climate suitability. The standard deviation of each pixel was used to compare results from different algorithms and map uncertainty. As the range of values is different for each algorithm, outputs were converted to binary (0 and 1) by applying a sensitivity and specificity maximisation threshold, and maps were inspected for areas of disagreement. This threshold rule was chosen because it is objective, minimises both false positives and false negatives, and has been found to perform well in ecological niche models assessing the effects of climate change [19, 49].

Binary outputs were restricted to areas historically accessible to both species via dispersal (M area in the BAM diagram framework [50, 51]). The accessible areas of *L. intermedia* and *L. neivai* were delimited by adding a buffer of 100 km to the ecoregions where they occur (data from FAO GeoNetwork [http://www.fao.org/geonetwork]).

The performance of each model was evaluated by the true skill statistic (TSS), a derivative of Cohen’s kappa. While kappa alone is a popular measure of model performance, recent studies suggest that it is overly dependent on presence data, and equal proportions of presences and absences only contribute to the kappa score when sensitivity and specificity are uniform, which biases estimates of predictive accuracy [52, 53]. TSS scores range from -1 to +1, with +1 indicating complete agreement and values close to and below 0 denoting models no better than random predictions.

Only model outputs with TSS scores above or equal to 0.7 were retained for mapping the climatic suitability areas of *L. intermedia* and *L. neivai*. Outputs with the highest TSS scores from each algorithm were overlaid and areas of agreement extracted per the majority ensemble rule [54], to produce binary ensemble maps. These were validated by TSS using the set of presence records that was left out of the modelling procedures during the spatial thinning process (see above). Potential changes in the climatic suitability of *L. intermedia* and *L. neivai* were assessed from these maps.

### Predicting range changes of sand flies in response to climate change

The approximate area of climatic suitability was extracted from the final binary ensemble maps of each species under each scenario (current, RCP4.5 and RCP8.5), to give an objective numerical overview of potential climatic habitat contraction and expansion. Binary model predictions from each scenario were overlapped in ArcGIS v. 10.00 [30] to map each species’ current climatic range and potential future expansion and contraction. Areas of intersection between the two species under the different scenarios were also mapped in ArcGIS v. 10.00.

## Results

### Descriptions of the ecological niches of the two sand fly species

As explained in the Methods, a subset of six less correlated variables was selected for ecological niche modelling, to reduce collinearity in the initial dataset of 19 climatic variables. These six variables include mean annual temperature and precipitation as well as measures of diurnal (temperature) and seasonal (temperature and precipitation) climatic changes (Table 1).

**Table 1:** Climatic variables associated with presence records of *Lutzomyia intermedia* and *Lutzomyia neivai*

	*Lutzomyia intermedia*				*Lutzomyia neivai*				Difference^a^	
	Min.	Median	Mean	Max.	Min.	Median	Mean	Max.	W	*P*
Annual mean temperature (°C)	16.5	22.8	22.41	27.6	15.9	21.1	21.05	25.1	55,316	< 0.001
Diurnal range of temperature (°C)	6.3	10.55	10.57	14.2	7.2	12.3	12	15	19,292	< 0.001
Temperature seasonality (standard deviation)	4	19.56	19.48	33.44	2.93	28.83	36.66	50.98	10,558	< 0.001
Annual precipitation (mm)	601	1324	1368	2525	567	1345	1320	2013	38,144	0.918
Precipitation seasonality (coefficient of variation)	14	62	61.87	104	6	46	50.31	95	49,294	< 0.001
Precipitation of warmest quarter (mm)	18	488	497.3	948	198	487	490.5	782	39,243	0.489

^a^Statistical significance given by Wilcoxon rank sum tests (W)

#### Annual mean temperature versus annual precipitation

**Fig. 1:**
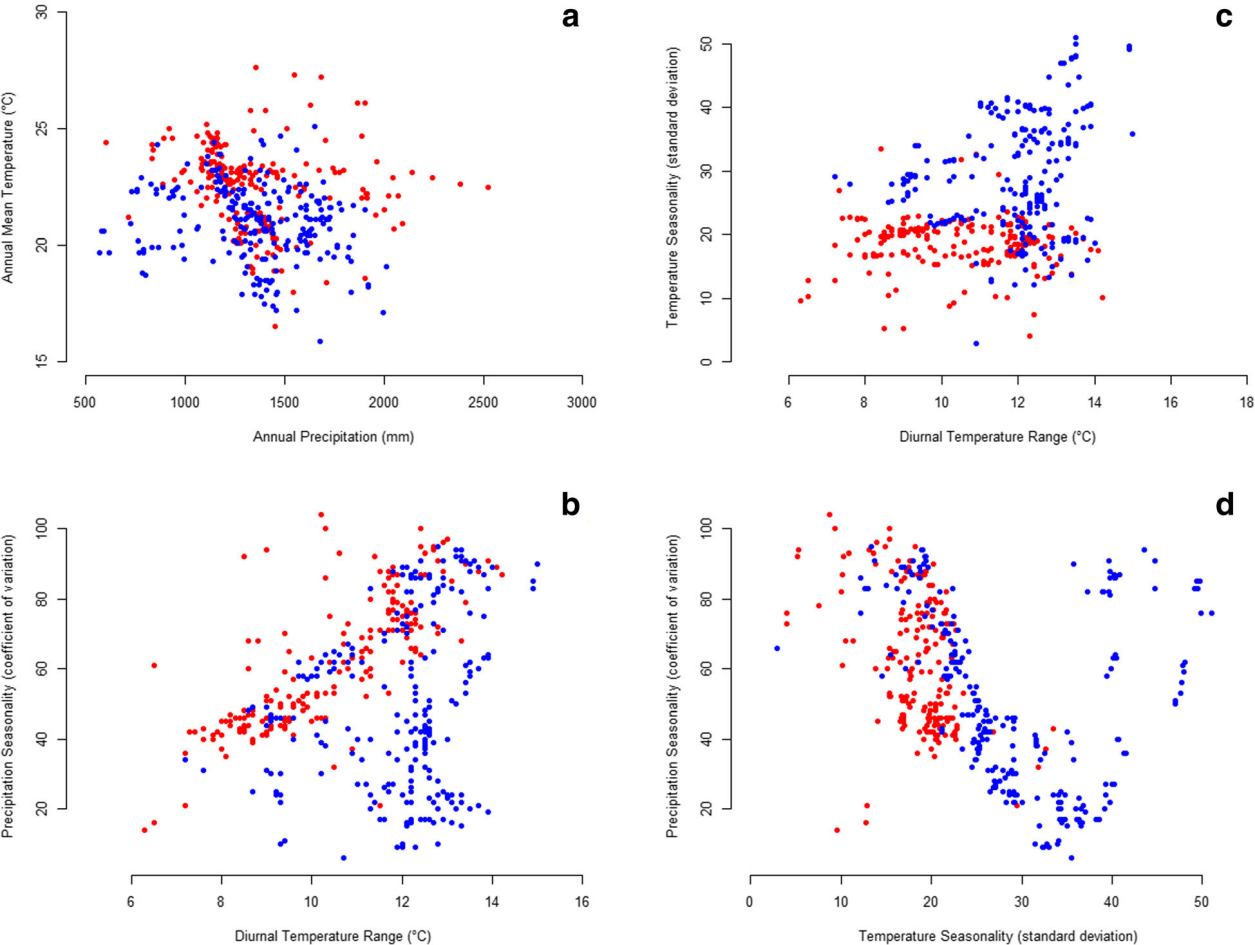
Bioclimatic variables of records of *Lutzomyia intermedia* (*red dots*) and *Lutzomyia neivai* (*blue dots*). **a** Annual mean temperature (°C) by annual precipitation. **b** Precipitation seasonality (coefficient of variation) by diurnal temperature range (°C). **c** Temperature seasonality (standard deviation) by diurnal temperature range (°C). **d** Precipitation seasonality (coefficient of variation) by temperature seasonality (standard deviation)

Annual mean temperature was higher for *L. intermedia* than for *L. neivai*, while the difference in annual precipitation was not statistically significant (Fig. 1, Table 1). Most records in the bottom left corner of the scatter plot (17.5–22.5 °C, 500–1,000 mm) are for *L. neivai* (27 out of 28); while most records in the top right corner of the scatter plot (22.5–27.5 °C, 2,000–2,500 mm) are for *L. intermedia* (7 out of 7).

#### Precipitation seasonality *versus* diurnal temperature range

Most records in the top left quadrant of the scatter plot (60–110 coefficient of variation, 6–11 °C) are for *L. intermedia* (17 out of 23); while most records in the bottom right quadrant of the scatter plot (10–60 coefficient of variation, 11–16 °C) are for *L. neivai* (83 out of 91) (Fig. 1, Table 1).

#### Temperature seasonality versus diurnal temperature range

Both mean temperature seasonality and diurnal temperature range were statistically much higher or higher, respectively, for *L. neivai* than for *L. intermedia*. Most records in the bottom left quadrant of the scatter plot (0–25 standard deviations, 6–12 °C) are for *L. intermedia* (80 out of 112); while most records in the top right quadrant of the scatter plot (25–50 standard deviations, 12–18 °C) are for *L. neivai* (43 out of 43) (Fig. 1, Table 1).

#### Precipitation seasonality versus temperature seasonality

Mean temperature seasonality, but not mean precipitation seasonality, was statistically much higher for *L. neivai* than for *L. intermedia*, with only the former occurring where temperature seasonality shows > 35 standard deviations. For *L. neivai,* precipitation seasonality displays a positive quadratic distribution with temperature seasonality > 10 standard deviations (all but one record) (Fig. 1, Table 1).

### Ecological niche models for the two sand fly species

**Fig. 2:**
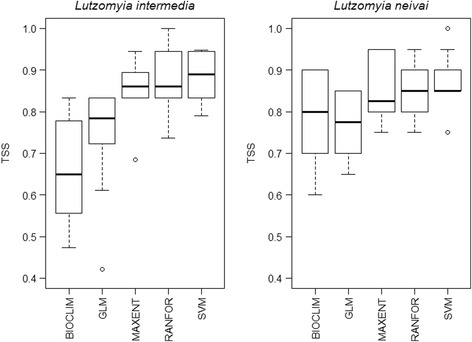
Model performance of different algorithms. *Abbreviations*: TSS, true skill statistic; GLM, generalised linear model, logistic regression; MAXENT, maximum entropy; RANFOR, random forest; SVM, support vector machines

**Fig. 3:**
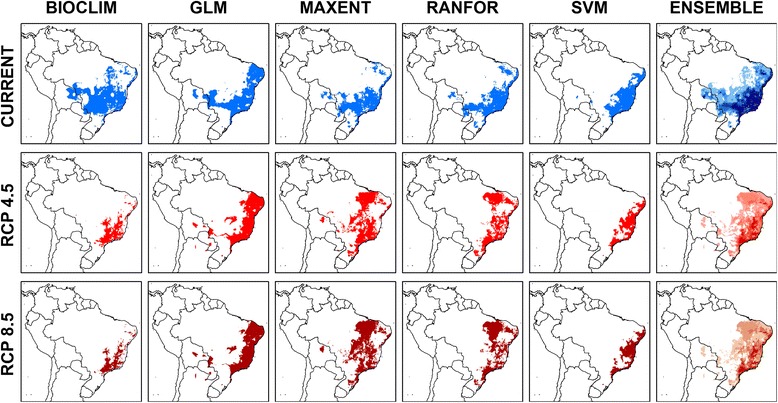
Current and future climatic suitability for *Lutzomyia intermedia* from five modelling algorithms. *Abbreviations*: GLM, generalised linear model, logistic regression; MAXENT, maximum entropy; RANFOR, random forest; SVM, support vector machines

Model performance ranged from good to excellent (0.6 < TSS < 1.0) for all five ecological niche modelling algorithms, with only BIOCLIM and GLM occasionally having mean TSS scores < 0.8 (Fig. 2). Model outputs agreed most in identifying south-east and northeast Brazil as having current climatic conditions suitable for *L. intermedia* (dark blue in current ensemble map of Fig. 3). A preliminary version of the models included unoccupied areas in countries to the west and north that were removed by restricting the results to accessible areas for the species.

**Fig. 4:**
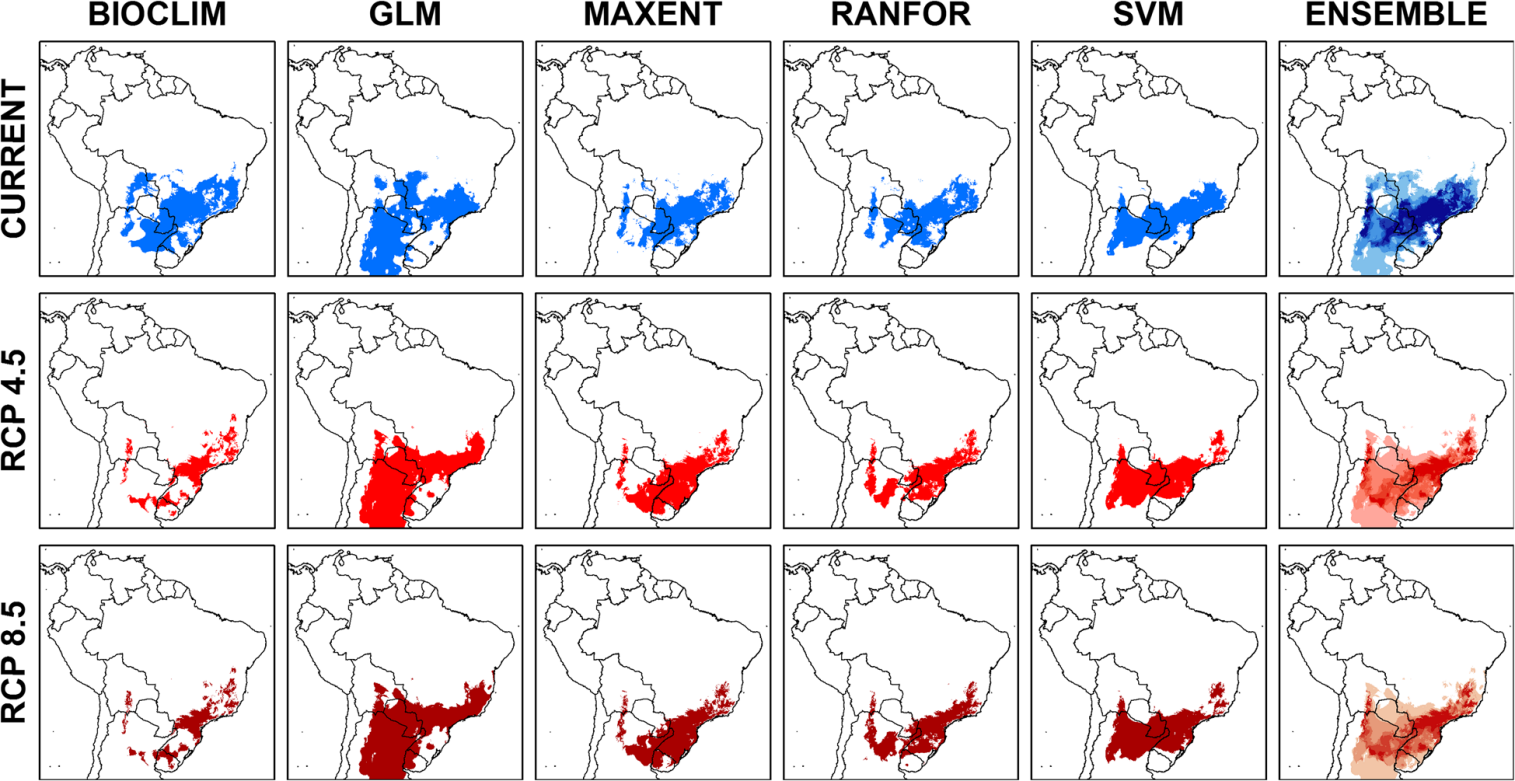
Current and future climatic suitability for *Lutzomyia neivai* from five modelling algorithms. *Abbreviations*: GLM, generalised linear model, logistic regression; MAXENT, maximum entropy; RANFOR, random forest; SVM, support vector machines

For *L. neivai*, the model agreement for current climatic conditions was higher in south-east/south Brazil, east Paraguay, northeast/north-west Argentina and a small area in southern Bolivia (dark blue in current ensemble map of Fig. 4).

### Predicting range changes for the sand fly species based on ecological niche modelling and two climate change scenarios

Both climate change scenarios (RCP 4.5 and RCP 8.5) predicted similar geographical distributions for the combinations of climatic variables identified by the ecological niche modelling algorithms as being suitable for *L. intermedia* (Fig. 3) and *L. neivai* (Fig. 4).

**Fig. 5:**
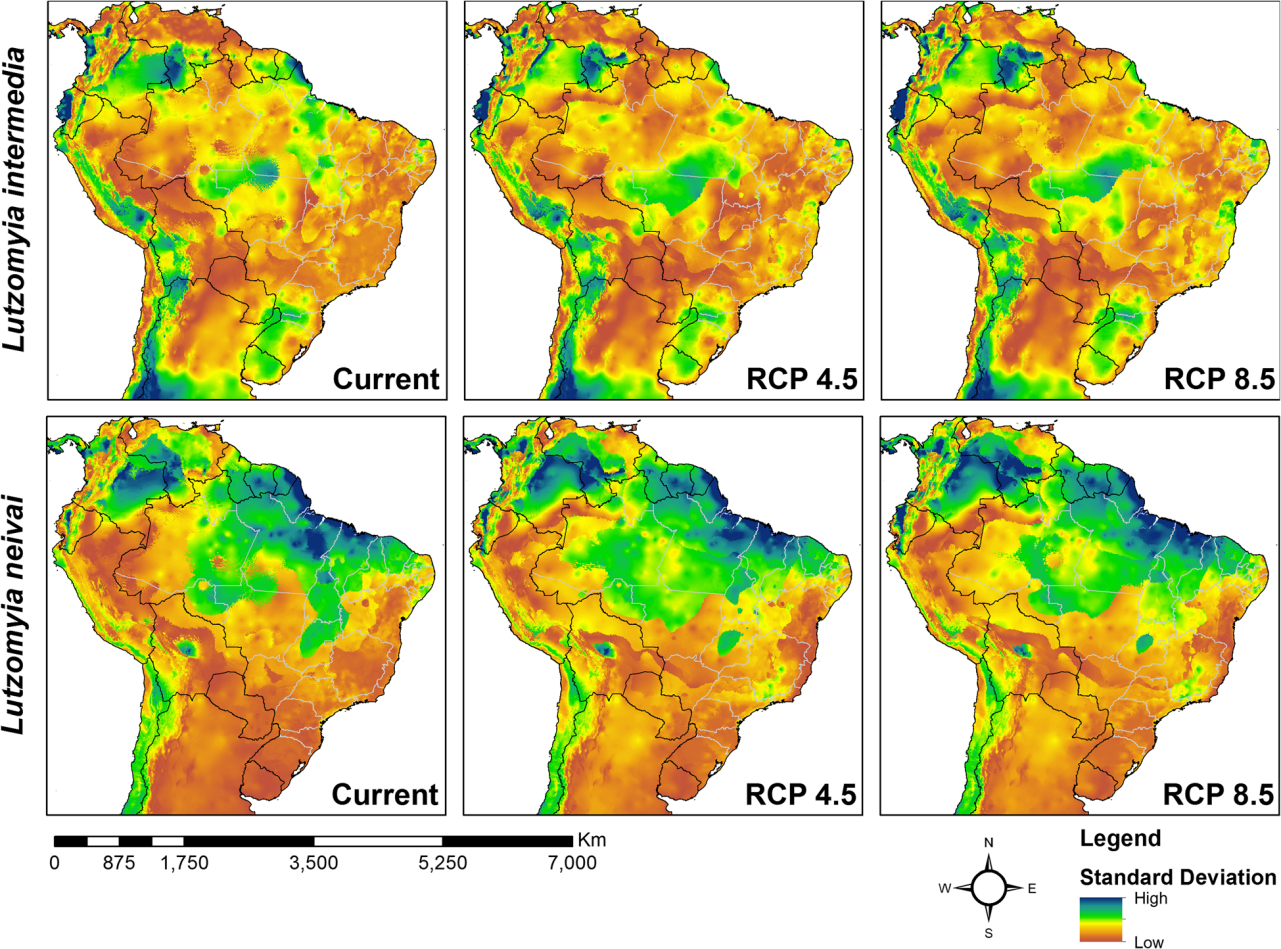
Uncertainty mapping for models of *Lutzomyia intermedia* (top) and *Lutzomyia neivai* (bottom)

For all ecological niche modelling algorithms, each climate change scenario predicted modifications in the distributions of the climatic conditions suitable for both sand fly species within the four countries where they currently occur, namely Argentina, Bolivia, Brazil and Paraguay. Uncertainty mapping showed the least confidence in current and future predictions for *L. intermedia* in the Andean mountains, southern Colombia, southern Venezuela and southern Amazonian Brazil (Fig. 5), and least confidence in current and future predictions for *L. neivai* in most areas north of its current range (Fig. 5).

**Fig. 6:**
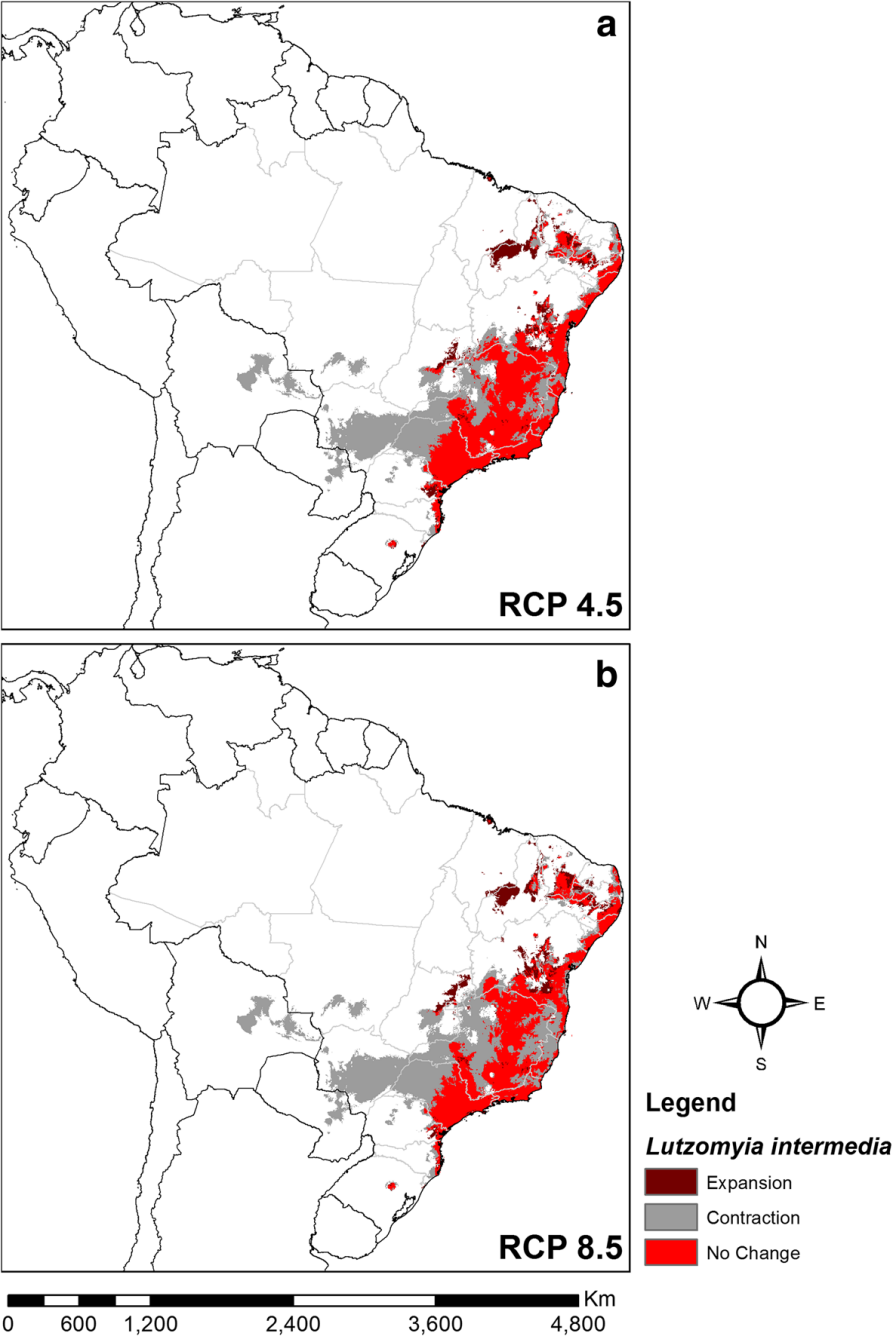
Consensus maps of future climatic suitability of *Lutzomyia intermedia*. **a** RCP 4.5 (stabilisation scenario). **b** RCP 8.5 (high emissions scenario)

**Fig. 7:**
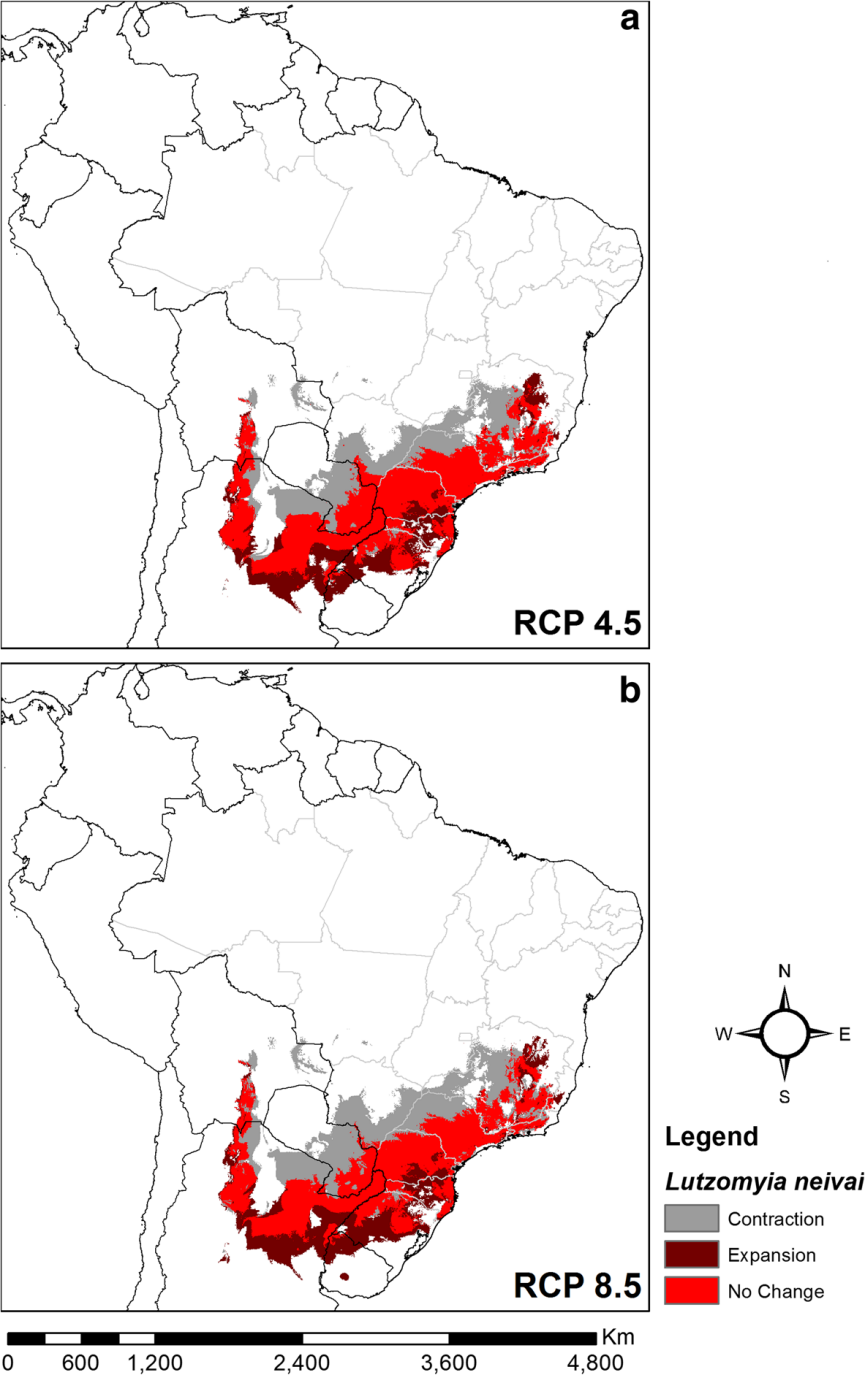
Consensus maps of future climatic suitability of *Lutzomyia neivai*. **a** RCP 4.5 (stabilisation scenario). **b** RCP 8.5 (high emissions scenario)

Using the consensus for the five ecological niche modelling algorithms, both climate change scenarios indicated different patterns of range stability, contraction or expansion for *L. intermedia* (Fig. 6) and *L. neivai* (Fig. 7). For *L. intermedia* the predictions were for substantial contraction in the southern part of its range, where uncertainty mapping lent confidence to the predictions; and any expansion was limited to small areas in the northern part of its range, where uncertainty mapping suggested moderate-high confidence in the predictions. In contrast, for *L. neivai* the predictions were for a large range shift southwards, and uncertainty mapping lent confidence to the predictions.

Overall for the two climate change scenarios, the range of *L. intermedia* was predicted to contract by 41.1% or 46.8%, and the range of *L. neivai* was predicted to contract by 14.8% or 16.2% (Table 2).

**Table 2:** Predicted current area of climatic suitability for *Lutzomyia intermedia* and *Lutzomyia neivai* under two climate change scenarios (RCP 4.5 and RCP 8.5)

	*Lutzomyia intermedia*		*Lutzomyia neivai*	
	Total (km^2^)	Difference (%)	Total (km^2^)	Difference (%)
Current	1,958,675		2,179,175	
RCP 4.5	1,154,625	-41.1	1,857,600	-14.8
RCP 8.5	1,041,700	-46.8	1,825,475	-16.2

**Fig. 8:**
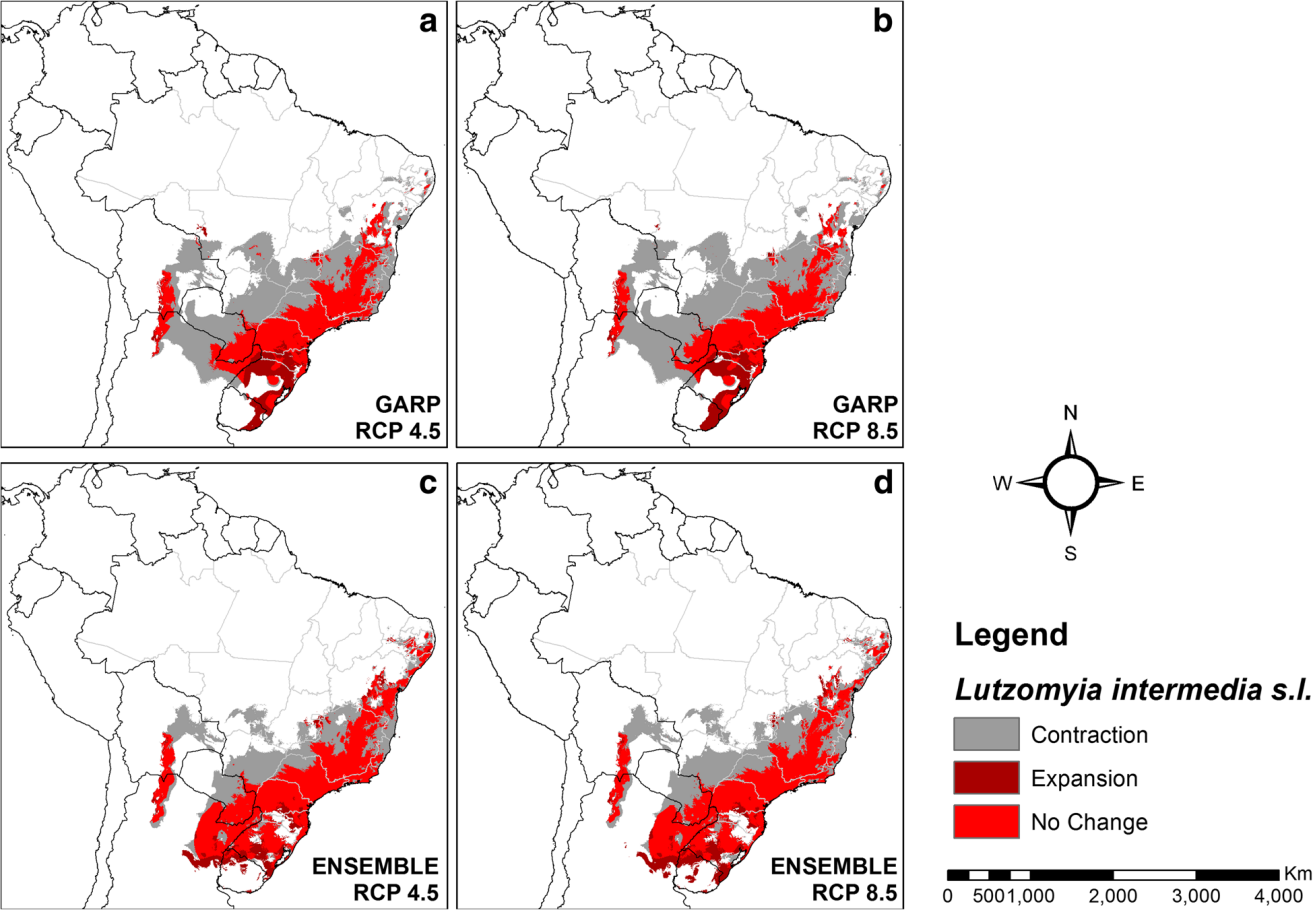
Comparison between GARP and ensemble models of *Lutzomyia intermedia (s.l.)*. **a**, **b** Models produced by GARP. **c**, **d** Models produced by ensemble of five algorithms (BIOCLIM, GLM, MaxEnt, RANFOR and SVM)

Peterson and Shaw [16] used the algorithm GARP to model the ecological niche of *L. intermedia *(*s.l*.) and, in the current analysis, it provided similar predictions to our ensemble models for both climate change scenarios when a selection of pre-2002 presence records was combined for both species (Fig. 8).

## Discussion

### Potential climatic niches under climate change scenarios

*Lutzomyia intermedia* was shown to be the more tropical of the two species, with its climatic niche being defined by higher annual mean temperatures, lower temperature seasonality and sometimes higher precipitation seasonality (Fig. 1). In contrast, *L. neivai* was shown to be more subtropical, and diapause [4] might allow it to survive very high-temperature seasonality sometimes associated with lower precipitation seasonality. The ability of *L. intermedia* to survive in warmer and more humid environments than *L. neivai* was previously suggested [28]. Differences in latitudinal ranges explain the predicted responses of the two species to climate change, with the tropical *L. intermedia* mostly contracting its range (Fig. 6), and the subtropical *L. neivai* mostly shifting its range southwards (Fig. 7). This is a common difference between tropical and subtropical species, probably resulting from their adaptations to natural climate change in previous geological periods [43].

### New predicted species distributions differ from those made in 2003

Peterson & Shaw [16] previously published an ecological niche model for *L. intermedia *(*s.l*.), but not for each of the two species owing to the paucity of confirmed presence records at the time. Based on a GARP model, Peterson & Shaw [16] found that environments suitable for *L. intermedia *(*s.l*.) extended from Rio Grande do Norte and Ceará states in northeast Brazil, south along Brazil’s eastern coast into Uruguay and Argentina, and west into Paraguay, Bolivia and Peru, with small disjunct pockets in Colombia and Guyana. Using two climate change scenarios, HHGSDX50 (conservative) and HHGGAX50 (extreme), they predicted *L. intermedia *(*s.l*.) will experience slight climatic improvements in its current habitats, and spread further along the eastern slopes of the Andes [16].

There are several potential explanations for the differences in our findings and those of Peterson & Shaw [16]. First, the latter used only the GARP program, rather than the ensemble approach utilised in this study. This new approach significantly reduces the prediction uncertainty from the use of a single algorithm [25, 54]. Additionally, the dataset they worked from could not account for sand fly surveys conducted post-2001 and therefore had fewer records. Consequently, the significance of relationships between predictor and response variables may have been misinterpreted. To test the impact of these differences, we removed post-2001 studies from our dataset and applied the GARP algorithm to the reduced list (Fig. 8). The results were similar for the GARP and ensemble analyses, but neither predicted the pattern of range expansion reported in 2003 [16].

In a comparative study of the performance of five modelling techniques, Elith & Graham [24] found GARP was consistently outperformed by the newer methods, a result consistent with the findings of Peterson et al. [55]. In particular, it was prone to over-predicting the test species' distribution and had relatively low sensitivity and specificity scores. Therefore, the updated methods and dataset used in the present study are likely to have produced more accurate predictions of the current and future climatically suitable ranges of *L. intermedia* and *L. neivai*.

Additionally, the resolution of current and past climate data utilised by Peterson & Shaw [16] was coarser than it is in the present investigation, at five arc minutes rather than 2.5. While extremely high-resolution environmental data layers are not required for ecological niche modelling based on climate data, finer spatial data can capture environmental variability, particularly in mountainous areas, that can be obscured at coarser resolutions [34]. When comparing ten arc minutes and 30-s resolutions, Hijmans et al. [34] observed significant variation in climate predictions for some regions despite the overall agreement.

Again considering resolution, Peterson & Shaw [16] utilised scenarios from the HadCM2 coupled climate model, which has a much coarser resolution than the HadGEM2 predictions used in the present study (417 × 278 km at the equator compared to 208 × 139 km) [56]. HadGEM2 also accounts for more climate change processes, including modifications to vegetation through a dynamic vegetation layer, and has demonstrated significant improvements in predictive ability on previous Met Office climate models [57]. If vegetation type and land cover are regulating the dispersal of *L. intermedia,* as they appear to be for *L. neivai* [18], then the addition of the dynamic vegetation layer in HadGEM2 may have been particularly important in allowing the ecological niche models constructed in the present study to recognize limits to expansion due to climate change.

### Implications for predicting the establishment and maintenance of CL transmission

There is sufficient evidence to treat both *L. intermedia* and *L. neivai* as incriminated vectors of CL, with natural infections of *Leishmania* detected in São Paulo (*L. intermedia *(*s.l*.) [58, 59]), Rio de Janeiro (*L. intermedia*, [10, 60]), Espírito Santo (*L. intermedia* [61]), Paraná (*L. intermedia *(*s.l*.) [62]), Santa Catarina (*L. neivai* [63]); Rio Grande do Sul (*L. neivai* [60]) in Brazil, as well as Tucumán and Salta (*L. neivai* [12]) in Argentina.

**Fig. 9:**
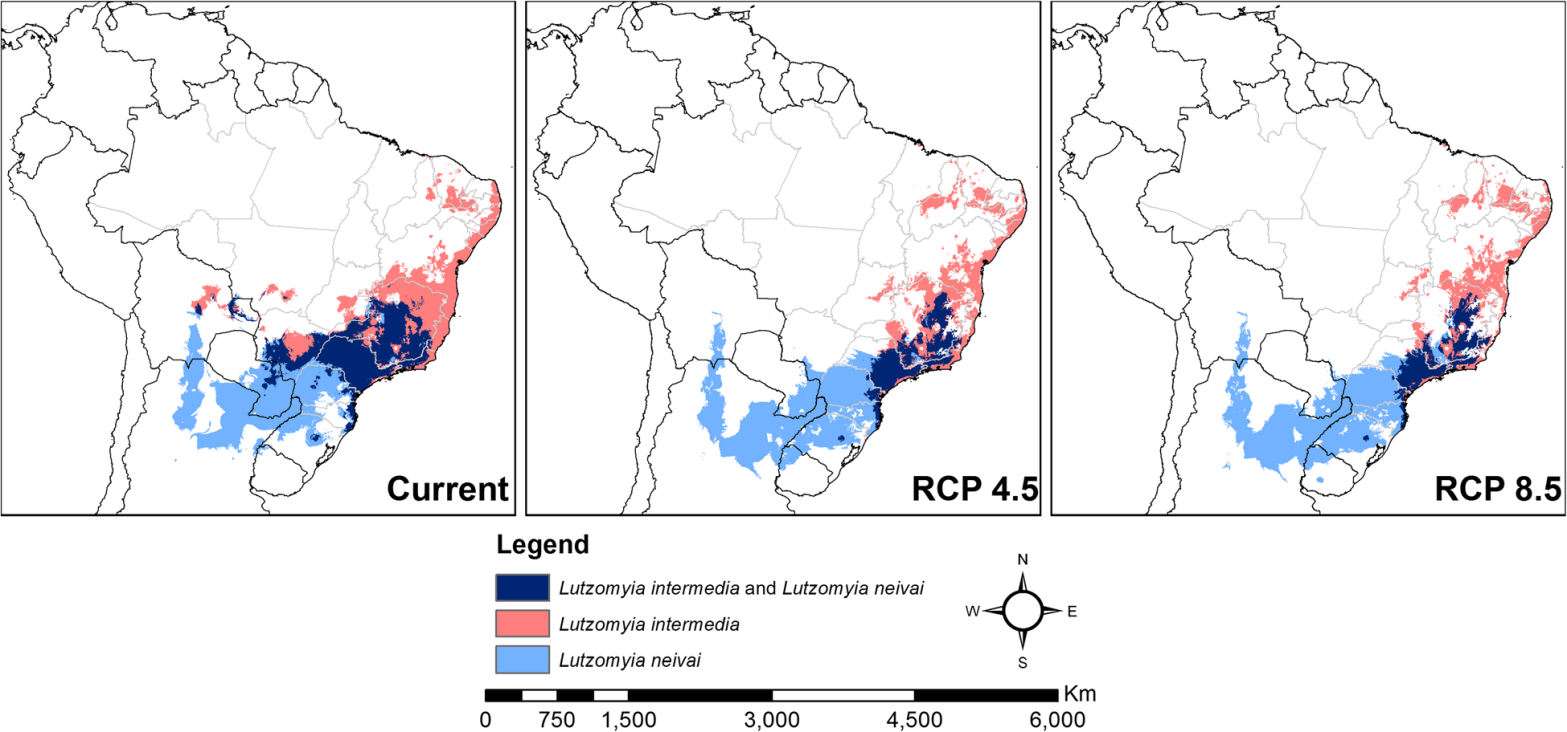
Intersection of model predictions for *Lutzomyia intermedia* and *Lutzomyia neivai*. Current and future (RCP 4.5 and RCP 8.5) predicted climatic suitability for *Lutzomyia intermedia* (*pink*) and for *Lutzomyia neivai* (*light blue*). *Dark blue* areas are predicted as suitable for both species

The intersection map (Fig. 9) shows that the range overlap between the two sand fly species in south-eastern Brazil will decrease substantially under both climate change scenarios. Currently, both species occur in sympatry mainly in the Brazilian states of São Paulo and Minas Gerais. Both species have been found in the Ribeira Valley, a CL endemic area in São Paulo, although substantial local variations in their densities have been reported: At Parque Estadual do Alto Ribeira (PETAR), an Atlantic forest reserve, their low densities suggested a minimum risk of disease transmission [64]; while in the nearby Serra district, where visiting tourists to PETAR stay, *L. intermedia* and *L. neivai* had such high frequencies that their presence in peridomestic areas suggested a high risk of CL transmission [65]. In Corinto and Lassance (Minas Gerais), the two species represented 97% of the captured sand flies and were suggested as local vectors [66]. The role of both species as CL vectors in sympatric areas thus might depend on local variations in population density determined by other environmental variables besides climate. Man-made environmental impacts such as deforestation might favour the selection of sand fly species that can survive in these areas [4, 9]. This was clear in the studies mentioned above in Ribeira Valley, where although forest and anthropic areas have very similar phlebotomine faunas, the frequency of *L. intermedia* and *L. neivai* was considerably higher in anthropic areas [64, 65]. Similar findings were observed in an ecotourism area of Rio de Janeiro, where *L. intermedia* predominated in peridomestic areas [67]. Climate may thus constrain the distribution of these species at coarser spatial scales, but other variables gain importance at fine scales, such as land use and cover [39]. The future loss of climate suitability in sympatric areas of *L. intermedia* and *L. neivai* might influence local changes in the distribution of both species and in the transmission of CL.

Our models indicate that *L. intermedia* will become more tropical, while *L. neivai* will shift southwards and become more subtropical. This contradicts the suggestion that *L. whitmani* might replace *L. intermedia *(*s.l*.) as the more important vector of *L. braziliensis* in southern Brazil and nearby Argentina [16]. Currently, *L. whitmani* is likely to share transmission of CL with *L. intermedia* only in the south-east region of Brazil, such as in Espírito Santo and Minas Gerais [9]. In fact, there is no evidence in the past 13 years that *L. whitmani* has spread into the far south of Brazil, where *L. neivai* is the only reported vector [4, 63]. Our results agree with the previous niche model of *L. neivai* produced by Maxent for north-west Argentina [18]. In Argentina, *L. neivai* is the most abundant sand fly species in CL transmission areas [68], and modelling predicts it will persist there in the future.

In northeast Brazil, the models predict a future increase in climatic suitability for *L. intermedia* mainly in central Piauí state. This is a region of transition between the Cerrado and Caatinga biomes, which are substantially drier and warmer than the Atlantic Forest where *L. intermedia* currently occurs [9]. This region is poorly sampled for sand flies. However, captures performed in the late 1990s detected only *L. longipalpis* and *L. whitmani* as potential vector species [69]. There is a single record of *L. intermedia* in Piauí State, in the capital Teresina [13, 28]. Future field studies should survey this area for the occurrence of *L. intermedia* and other potential vectors.

The shifting distributions of *L. intermedia* and *L. neivai* in response to climate change will affect regional investigations of transmission cycles, including those using overlaid ecological niche models of *Leishmania* and its vectors in south-east Brazil [21]. Because climate is a first order determinant of the spatial distribution of species [46, 70], predictive models at finer spatial scales are needed to guide more precise assessments of disease risk and surveillance. These models will require the inclusion of environmental variables at higher resolution, including land cover as well as climate.

## Conclusions

Our findings indicate that climate change will not always lead to the expansion of the geographical distribution of disease vectors such as sand flies. *Lutzomyia intermedia* and *L. neivai* will have smaller areas of climatic suitability available to them in the future, but they might disperse into new areas, such as southwards into Brazil and Argentina (*L. neivai*) and within northeast Brazil (*L. intermedia*). Ecological niche models should be species specific, carefully selected and combined in an ensemble approach.

## Additional file

Additional file 1: Table S1. Compiled presence records of *Lutzomyia intermedia* and *Lutzomyia neivai*. (XLSX 54 kb)

## Abbreviations

CL: Cutaneous leishmaniasis
CP: Representative concentration pathway
GARP: Genetic algorithm for rule set prediction
GLM: Generalised linear model
IPCC: Intergovernmental Panel on Climate Change
MaxEnt: Maximum entropy
RANFOR: Random forest
SVM: Support vector machines
TSS: True skill statistics
